# Unsung voices of technology in school education-findings using the constructivist grounded theory approach

**DOI:** 10.1186/s40561-021-00182-7

**Published:** 2022-01-04

**Authors:** V. Deepa, R. Sujatha, Jitendra Mohan

**Affiliations:** 1 Amity Business School, Amity University, Amity University Campus, Sector – 125, Noida, Uttar Pradesh 201313 India; 2 CIO and CISO, Coforge Ltd., Noida, Uttar Pradesh India

**Keywords:** School education, Constructivist grounded theory, Education technology, Socio-economic divide

## Abstract

Technology adoption for school education further gained momentum during the COVID-19 pandemic. However, the challenges and strategies of children belonging to the less privileged (we use ‘privileged’ in the article to identify those enjoying a standard of living or rights as majority of people in the society) families are different from those of the children who come from socio-economically better-off (privileged) backgrounds. The purpose of this research is to explore the experiences of children with school education and using technology for learning. Past studies have highlighted the use of internet and communication technologies as a promising solution to provide quality school education in the remotest parts of the country. Previous research has also ascertained that the socio-economic status divide has no significant impact on the students’ ability to learn using technology. Children can use technology to learn irrespective of their socio-economic status and background. We conducted this exploratory qualitative study from a constructivist grounded theory perspective. A purposive sample of 14 students (9 from underprivileged and 5 from privileged family backgrounds) in the age group of 6–14 years, was used and unstructured interviews were conducted. We analysed the data using constructivist grounded theory methodology. We found that the experiences of privileged and underprivileged children differed with respect to access to internet, affordability of ICT device, quality teachers, parental support, and financial sponsorship. However, the experiences and perspectives of the children were found to be similar with respect to personal ownership of mobile phone device for unlimited time at own disposal, self-directed learning and having a trusted study advisor. The findings may be useful to policy makers and EdTech firms to build strategies and solutions for effective implementation of universal school education in the country.

## Introduction

With millions of children currently out of school, the challenge of increasing enrolment, retention and learning in schools has opened up an opportunity to take a new, creative and transformative approach to thinking about education across the world (Birdsall et al., 2005). The COVID-19 crisis forced governments to explore the possibility of open and distance learning by adopting different digital technologies thereby increasing the confidence of the stakeholders towards adopting technology for school education (Jena, 2020, p. 19). There are many reasons brought out in favour of using Internet and Communication Technology (ICT) for Education in previous studies. ICT devices like phones, laptops, notebooks and tablets are able to host learning content and apps at no or minimal costs and gained popularity as crisis tools for learning during the pandemic (Naresh, 2020; Onyema et al., 2020). Interactive learning content, grounded by scientific learning theories are providing personalized and distance agnostic learning solutions, making it an attractive proposition for the society. Besides, ICT, owing to its reach and connectedness, can be used for changing the education scenario in rural India (Chatterjee et al., 2020; Jamir et al., 2019; Nedungadi et al., 2018). When a third of the world is living with no access to printed knowledge, skills, and technologies (Dhanarajan, 2009, p. 46), the massive penetration of mobile and internet technology opens new opportunities for education and connectedness at wider scope and scale. However this choice is a problem for the underprivileged students in the country because of lack of required infrastructure for the same (Jena, 2020). The term ‘underprivileged’ refers to the disadvantaged section of people who are economically, socially, and locationally in an unfavourable situation compared to the rest of the population of the society (Kundu, 2019). This concern is recognized and there is significant focus, effort, and progress on the infrastructural development to support technology-based education. Specifically in India, Government of India’s NOFN (National Optical Fibre Network) project aims to provide broadband connectivity with adequate bandwidth to 250,000 Gram Panchayats of the country (Krishna et al., 2014; Kumar et al., 2015; Sharma & Pandey, 2015). The project aims that people living across remotest villages of India have equitable access to ICT applications like e-commerce, e-banking, e-governance, e-education, Tele-medicine and will generate new trade and employment avenues in the country. In research concerning development rates of nations, new metrices analysis have put forth positive correlation between ICT development index and Human Development Index in a country, further advocating the need for advancing ICT pervasion in the country (Pérez-Castro et al., 2021). These developments will strengthen the ICT infrastructure and further enable implementation of ICT enabled school education in the remotest parts of the country. While the value of using ICT for education was ascertained, the actual adoption of ICT for education gained further momentum due to the global pandemic in the year 2020. The Covid-19 pandemic broke the adoption inertia prevailing in the society for many EdTech offerings. It brought the schools and colleges across the globe resort to online and remote learning approaches to maintain continuity in education. The EdTech firms used this opportunity to industrialise online education with attractive incubation options for consumers (Al Lily et al., 2020). During the pandemic lockdown many learning apps were offered free for children to make use the time well, which in turn led an upsurge in the adoption of these tools (Mulenga & Marbán, 2020; Pandey & Pal, 2020). These experiences gained during the pandemic paved way to lot of new research and increased understanding about using technology in education. Thus, at the onset previous research suggests many reasons to consider ICT as a solution to implement universal school education in India. At the same time, it is also recognized that the socio-economic and digital divide prevailing in the country can be a roadblock to realizing the benefits of ICT usage for school education (Azubuike et al., 2021; Singh & Chanda, 2021; Tewathia et al., 2020). While ICT tools could aid the teaching–learning process for some, they could also introduce a new layer of inequality in education for the digitally excluded. Studies have highlighted the efforts that are already underway to bridge this divide (Hillier, 2018; Rao, 2005), thereby making ICT leverage a plausible solution for implementing universal school education.

In this study we adopt a constructivist grounded theory approach to explore and examine the perspective of the children about their experience with school education and using technology for learning. School going children are minors and we believe that to understand their reality it is important to engage with them empathetically and capture their subjective experience. We deemed the constructivist grounded theory approach as most suitable approach in this context, for this qualitative study. We find unapprised aspects, that we call the “unsung voices”, of technology in school education in the form of students’ perspectives through this approach. The study enables us to represent the children’s perspective of school education through a framework. The findings may be useful to policy makers and EdTech firms to build strategies and solutions for effective implementation of universal school education in the country.

### Problem statement

Most of the related studies in the past are directing towards making ICT enabled school education a universal reality in the times to come. It becomes imperative, in this context, to understand the perspective of the primary stakeholder and consumer of these initiatives i.e., the children themselves. However, we could hardly find any research that focused on exploring and examining the perspectives of the children about school education, particularly with respect to how they learn for school and use ICT for learning. Some studies have been conducted in related areas that examine the student perspective of online education in higher education institutions (Phutela & Dwivedi, 2020; Vizo et al., 2020). Previous studies concerning school students have examined teaching methods, online education, returning to school and school environment (Backman et al., 2012; Harvey et al., 2014; Mealings et al., 2012; Phutela & Dwivedi, 2020). However, no study has been conducted with a constructivist grounded approach to explore school students’ experiences with school education and how these differ for students who come from socio-economically weaker section of our society to the best of our knowledge.

### Purpose of the study

The purpose of this research is to explore the experiences of children with school education and using technology for learning. Particularly with respect to how they learn for school, the challenges they face, the strategies they adopt, how they use ICT (Internet and Communication Technology) tools for learning. We examine how these experiences differ for children coming from socio-economically weaker sections in the country. We use the constructivist grounded theory approach and represent the children’s perspective of school education through a framework.

### Objectives of the study

- To examine the children’s perspective about the challenges and learning strategies they adopt for school studies.
- To examine how the experiences of children coming from underprivileged backgrounds is distinct from those of the children from the better-off (privileged) families.
- To develop a theoretical framework for understanding experiences of children with school education and use of technology for learning.

### Contributions of the study

Past studies have highlighted the use of internet and communication technologies as a promising solution to provide quality school education in the remotest parts of the country. Previous research has also ascertained that the socio-economic status divide has no significant impact on the students’ ability to learn using technology. Children can use technology to learn irrespective of their socio-economic status and background. While most of the existing literature is directing towards leveraging technology as a promising solution for implementing universal school education, we could not find any open-ended research that could examine the phenomenon from the student’s perspective, particularly to examine how really children learn during school, if, and how they use ICT for the same. Past literature, though, acknowledges that the socio-economic divide creates distinct experiences for children from underprivileged families with respect to school education, we could not find any study that examined the perspectives of students from both categories (privileged and underprivileged) to see how different or similar they were.

With this study, we add to the existing literature in technology in school education, by capturing children’s perspective on school education and learning into a theoretical framework. We verify findings from previous research by corroborating it with the perspective of the primary stakeholders and consumers of these initiatives namely the children and identify new themes (Trusted study advisors, Self-directed learning, and Personal ownership of device) for future exploration in this area. The present study also brings out the distinct experiences, challenges and learning strategies of children from privileged and underprivileged families.

### Research questions

- What is the children’s perspective about the challenges and learning strategies adopted by them for school studies?
- How are the experiences of children coming from underprivileged backgrounds distinct from those of the children from the better-off (privileged) families?

## Literature review

### Children adopting technology in school education

The COVID-19 pandemic brought with it a momentous change in the life of school going children across the globe. To maintain continuity in learning, schools and teachers rapidly adopted distance learning using resources from global actors (Gavrielatos, 2020)**.** High relevance leads to high adoption of EdTech (Kiran et al., 2020), the pandemic led to increased relevance and thereby increased adoption for EdTech offerings. However, connectivity is a restrictive factor in adoption of EdTech (Tobin & Hieker, 2021), thereby created different experiences for children who were devoid of requisite tools and infrastructure to avail online education. Previous research indicates that after the COVID-19 experience, students are ready to adopt online learning environments, though connectivity and bandwidth issues are among the biggest barriers for children in rural areas (Muthuprasad et al., 2021). Students have a positive perception about using technology to learn, however age, gender and academic background affect their level of confidence for instance male are more confident than females, science and maths students are more tech savvy, elder students are less confident than younger students (Kahveci, 2010). The new EdTech tools make education more effective, affordable, connected to the circumstances of specific students (Zeide, 2016). With the increase in costs for providing education and concerns about financial responsibility, elevated awareness of teacher skills and students learning styles and needs, more focus is being placed on promises offered by online software and educational technology (Regan & Jesse, 2019). At the same time, it is also recognized that the perception of parents, teachers, access to infrastructure, pedagogical standards used, relevance and learner motivation are critical to children adopting EdTech (Tauson & Stannard, 2018). Technology infusion in teaching changes teachers’ perception from “learning technology” to “using it to support learning for students” (Vannatta et al., 2001). For new school technology to be successful, significant planning, teacher training and resources must be in place (Tyler-Wood et al., 2018). Children in school are confident and open to use technology and training them on reading, writing and research skills will allow for harnessing this (Watson, 1998). Studies have been conducted using experimental methods to capture children’s perception about using technology for learning (Kahveci, 2010; Rennie & Jarvis, 1995; Zhao et al., 2019). Studies have also highlighted the importance of other stakeholder perceptions like that of the parents and teachers in leveraging technology for children education (Lee et al., 2014; Olmstead, 2013). Methods like observation and video recording have been used. Studies have focused on ability of students to learn using technology and used observational or experimental techniques to support development of effective EdTech solutions. Focus has been extended to study such ability in students belonging to economically weaker backgrounds to uplift their educational experiences. Most of the studies indicate towards children being naturally inclined to learn using technology irrespective of their socio-economic background if they were provided with requisite infrastructure and device. Blended learning platforms in classroom settings with quality digital content, expert online teachers, and on-site teaching assistants can improve well-being and learning achievements of students in school irrespective of their socio-economic status (Dey & Bandyopadhyay, 2019).

### Children’s perspective about school education

Including children in the identification and exploration of issues important to them promotes a positive sense of inclusivity and such approaches to developing pedagogies of citizenship and belonging constitute a practical enacting of ‘voice’. Children’s voices are central to any study of their perspectives (Nutbrown & Clough, 2009). Extensive research and progress is evident towards using technology in school education. However, we found that most of the recent studies related to school education are polarized around ICT adoption, almost leading to a prescriptive direction, without sufficiently examining the students’ perspective, who are the primary consumers and proposed beneficiaries of these initiatives. We could not find studies that particularly examined the children’s perspective with respect to how they usually learned for school, whether they used technology, what challenges they faced while studying and what strategies they adopted to deal with those. Limited research has been conducted in different countries to explore children’s experiences with school education and have highlighted specific concerns. A study in Sweden examined the perspectives of children with different experiences (those with learning difficulties and were receiving additional support from school and those without learning difficulties) and found that it is important to elicit narratives from children with different experiences to understanding the characteristics and deficits of educational environments (Allodi, 2002). Another study further found that there were individual differences with respect to likes and dislikes in school and children do not find they have any influence on the school curriculum (Einarsdottir, 2010). The role of teacher and instructional methods is also an area sufficiently highlighted in past studies. Introduction of authentic activities into daily instruction, teacher scaffolding and strategy instruction are essential supports to success of class room tasks assigned to children (Turner, 1995). Relationship with teachers and peers has been emphasized during school years for children forced to live in out-of-home care (Townsend et al., 2020). There is little information about and understanding of how children from families living in poverty experience school as opposed to children from better-off families. Amongst them is from a study conducted based on views of children aged between 5 and 11 years in Northern Ireland that found that family poverty affects every aspect of child’s experience of school and therefore policy interventions to improve educational outcomes are unlikely to be effective unless they are accompanied by far broader social policy initiatives (Horgan, 2009). Formal school is viewed as important for equipping children with skills to increase opportunities coming from socio-economically weaker sections of the society. Previous study suggests that children from under privileged backgrounds are balancing expectations for the future with responsibilities to their families in the present (Morrow, 2013). But none of these studies particularly examine from the children’s perspective what enabled their learning for school, ‘if’ and ‘how’ they used technology to learn, what challenges they faced and how they dealt with those.

## Research design

### Research approach

With an explorative purpose for this qualitative inquiry, we followed a constructivist grounded theory approach. Constructivist grounded theory allows data collection, data analysis and theory to stand in reciprocal relationships with each other. It follows an iterative process of constant comparison within and amongst data cases, theory and researcher field notes and memos (Charmaz & Belgrave, 2012; Corbin & Strauss, 1990; Gordon-Finlayson, 2010). School going children are minors and we believe to understand their reality it is important to engage with them empathetically and capture their subjective experience. Therefore, we deemed the constructivist grounded theory approach as most suitable for this qualitative study. Subjective experience is real and socially constructed (Terre Blanche & Kelly, 1999). Constructivist grounded theorists see meaning as mutually constructed between the researcher and the researched (Charmaz & Belgrave, 2012). Therefore, we do not claim any neutrality in our process of making meaning here, however we have tried our best to use rigorous data analysis strategies and to report on these.

### Research strategy

In the context of the objectives and the aim to develop a theoretical framework based on the views of the participants, multiple case study method, together with grounded theory data collection and analysis was our research strategy. Evidence created from multiple case studies is strong and reliable, difference and similarities between the cases can be understood, contrasts and similarities provide strong influence to literature, more convincing theory is created as suggestions are grounded in several empirical evidence (Gustafsson, n.d.). Fourteen individual case studies, together with grounded theory data collection and analysis was the method used to conduct this research. We constantly compared data excerpts within and between the fourteen cases as well as with the relevant theory to the point of theoretical saturation, when we found no themes emerging. With theory development as a grounded theory directive, we developed a core theoretical understanding of the experiences of children.

### Research method

#### Sampling

Theoretical sampling directed the purposeful selection (cf. Babbie & Mouton, 2001) of 14 children 9 of whom belonged to underprivileged families and were studying in a local NGO. The criteria followed to recruit research participants was that they should be school going children. Since we wanted to particularly examine the distinct experiences of children from underprivileged and those from privileged family backgrounds, participants were recruited from both the clusters. In total 9 children from underprivileged and 5 from better off families took part. All the underprivileged children were recruited through ‘Bhavishya’, an NGO that sponsors school education for the children of migrant workers who earn livelihood as daily wage labourers. The remaining children were recruited through families known to the researchers. All the 14 children live in the National Capital Region (NCR) of India and were aged between 6 and 14 years at the time of the study. We added consequent follow-up interviews with the same 14 students. After our initial engagement with 7 students from underprivileged, we interviewed 4 students from privileged category. After analysis of the data, we approached two additional participants from underprivileged and one from privileged category, to confirm theoretical saturation. We allocated numbers to the research participants to ensure anonymity. Table 1 describes the research participants.

**Table 1:** Participant details

Research participant	Class grade	Gender	Family status	Occupation		School type
				Father	Mother	
RP1	8	Male	Underprivileged	Deceased	House help	Govt. Aided
RP2	4	Female	Underprivileged	Labourer	Maid Servant	Govt. Aided
RP3	5	Male	Underprivileged	Labourer	Housewife	Govt. Aided
RP4	6	Female	Underprivileged	Labourer	House help	Govt. Aided
RP5	6	Male	Underprivileged	Labourer	Labourer	Govt. Aided
RP6	5	Male	Underprivileged	Labourer	Labourer	Govt. Aided
RP7	1	Female	Underprivileged	Labourer	Labourer	Govt. Aided
RP8	3	Male	Underprivileged	Labourer	Labourer	Govt. Aided
RP9	2	Male	Underprivileged	Guard	Housewife	Govt. Aided
RP10	8	Male	Privileged	Mgmt.	Mgmt.	Global School
RP11	9	Male	Privileged	Trainer	Housewife	Private
RP12	7	Male	Privileged	Designer	Professor	Private
RP13	5	Female	Privileged	Business	Housewife	Global
RP14	4	Female	Privileged	Doctor	Doctor	Private

*RP* research participant, *Mgmnt* management, *Govt.* government

### Research setting

Nine participants belonged to underprivileged backgrounds. In assessing their suitability, we took help from “Bhavishya,” an NGO that has been supporting education of children of migrant labourers who come from villages of Northern India since 2016. The NGO coordinators helped us to conduct unstructured interviews with the identified children. Since the research participants were minors, due consideration was given to the ethical issues concerning research involving children. We took help from the NGO for NGO participant cluster. The NGO coordinator helped us to discuss the research purpose and ethical parameters like freedom to withdraw and confidentiality with each participant and their parents before the interview. The other five participants were from privileged families. We used convenience sampling method to identify these families. At least one of the child’s parents was known to any one of the researchers for all these participants. The criteria to recruit parents were that they had school going children. We briefed parents individually through a telephonic conversation or chat and took consent for their ward’s participation in the research. We shared the background for the research purpose as well as the ethical parameters. The participating children were made comfortable and briefed about the research purpose, before and reiterated during the interviews.

### Data collection

First, we conducted interviews with the children from the NGO. Some of them were not fluent in English, so we used both English and Hindi during the discussion. We used unstructured interviews to elicit the perspectives of the participating children with respect to their experience with school studies. Zoom tool was used for recording the interviews. A few follow up conversations were done telephonically with some participants. Questions of inquiry were based on the conceptualized issues identified during literature review. Questions were aimed at eliciting their perspective what helped them with studies in school, whether they used internet and apps for learning, what challenges they faced and how they dealt with those. We used comforting questions to get started like “tell me, do you enjoy school and studying? What do you most like?” As the discussion flowed from there, we used counselling skill conventions, like paraphrasing, probing and reflection, to develop the discussion in line with the research context and objective. Care was taken to keep the questions open-ended so that the children can raise and talk about the issues that are important to them. The leading interview questions are mentioned below.

- “How do you study usually?”
- “When you want to learn something new, what do you do?”
- “When you do not understand something that you are trying to learn, what do you do?”
- “Do you use the internet?”
- “Do you use the internet for studies?”
- “Do you have your own mobile phone or computer?”
- “Have you used any mobile app for learning?”
- “tell me about something you learnt on your own? What did you do? How did you learn?”
- Subsequently, from a theoretical sampling standpoint, we conducted follow up interviews individually as well as in groups, to elaborate or confirm emerging themes and meanings.

### Recording of data

The interviews were recorded digitally using the zoom tool and were substantiated with field notes. Later one of the authors transcribed the interviews verbatim and prepared the data for analysis. We transcribed the interviews verbatim to ensure immersion in the data.

### Data analysis

We used coding and memoing as the primary grounded theory analytical conventions. We used open coding—line by line, sentence by sentence, several phrases, para by para, whole document and co-constructed the data with the interviewee. In-vivo codes (words used by the students) were picked from the narratives of the interviewees. Particular attention was paid to the language of the interviewees. To minimize the bias in coding, the memos were reviewed by all the three researchers independently and theoretically plausible concepts were chosen to move ahead. The data was broken up to examine what it constitutes, the conditions in which it emerged to identify themes/categories.

We analysed the interview transcription of RP1 line-by-line with each data piece (line, sentence, or paragraph) which we labelled according to its significance in relation to the research objectives. From these initial codes, we identified recurring themes that were re-labelled and categorized following a colour-coding process. We followed the same process for analysing the subsequent interviews. The coding and categorization process refined with every subsequent interview transcription that we analysed. We noticed recurring themes as we compared codes within each data set and across data sets. We wrote memos and recorded our interpretations of the meanings underlying the codes and themes through-out the analysis process We added new themes as they emerged till, we noticed a point of saturation which was in the sixth and seventh underprivileged student transcriptions and the third and fourth privileged student transcriptions. Some codes that initially seemed different also started to make sense in relation to one another, collapsed to build a conceptually broader theme and showed saturation. Causal and consequential relationships between the categories of data was used to combine the categorized data into themes. As we continued writing memos with our understanding and interpretations of the codes and evolving themes, they became more conceptually significant and useful to be recorded as findings. We paid particular attention not to apply any theoretical concept to the data in the beginning of the analysis. However, we believe our preconceived notions, personal experiential and theoretical perspectives affected our interpretations. The analysis progressed into a conceptual framework consisting of two main themes with explanatory sub-categories under each.

Constructivist grounded theory study results in a core theoretical idea as an outcome (Charmaz & Belgrave, 2012). The conceptual framework we propose, as an outcome of this research, presents our construction of children’s experience with school education and use of technology for learning.

### Ethical issues

The study aims at contributing towards improvement of policies and strategies towards universal school education. Since children’s voice was crucial for evidence generation in the context of the study, initial considerations were made for involving children. Information required from the children was not sensitive by nature and we found no reason for the leading interview questions to seem offensive or embarrassing to the participants or have any lasting impact on them. Considering that while researching children, adult researchers have the power to interpret data in any way that they please, we adopted the constructivist grounded theory methodology (Morrow & Richards, 2007). We attempted to stay congruent to the methodological and epistemological notions that underlie constructivist grounded theory research in our planning, data analysis and reporting of the study. We base the rigour and credibility of the research on the detailed description of the research methodology. Informed consent was taken from parents and the participating children. Additionally at the beginning of each interaction participants were informed that they could withdraw easily at any time during the interaction (David et al., 2001). This was necessary as young children can easily consent to involvement in something a trusted adult (like a parent or a teacher) encourages them to become involved in. None of the children chose to withdraw and happily participated in the interactions. Information is recorded in the study in a manner that the identity of the participants cannot be readily ascertained.

## Findings

In this section we describe the main themes and sub-themes in detail. The verbatim extracts from the data ground the findings. The proposed conceptual framework and the integration with relevant literature that was done after the data analysis is presented in the following section. Two main theme clusters emerged to structure the data, from the grounded theory analysis. The Individual Human Interventions and the Systemic/Technology Interventions that support students studying in school, according to the children’s perspective. Their primary sub-themes follow.

### Theme cluster: individual human interventions that support school children with studies, from the children’s perspective

1. Quality teacher
2. Parental support
3. Trusted study advisor
4. Self-directed learning

### Theme cluster: systemic/technology interventions that support school children with studies from the children’s perspective

1. Affordability of ICT device
2. Access to internet
3. Mobile phone personal ownership
4. Financial sponsorship/aid

### Theme cluster: individual human interventions that support school children with studies from their perspective

#### Quality teacher—person who teaches the subject in the institution

Children from both privileged and underprivileged family backgrounds recognize a significant role of their teacher in their studies. As evident from RP1, RP2 and RP11’s narrative extract in Box 1, teachers help students with their doubts. RP2 mentioned that when she wanted to learn dance, she went to a dance teacher. She recognized the need for a quality teacher to physically visit the dance class (Refer RP2 response extract in Box 1). They make the subject interesting and understandable (refer response extract of RP5, RP14, RP13 in Box 1). Helpful teachers are approachable, and students find their subject more interesting. Since students in the centre seemed to like to maths, we checked with the coordinator if there could be any reason. As can be seen in the NGO coordinator’s response extract mentioned in Box 1, the interest in maths may be associated with the teachers being good in maths owing to their engineering background. While children from both categories recognized the need of a quality teacher for studies, their experiences differed with respect to access to teachers. NGO children were enrolled in nearby municipal corporation school, and they found that their teachers at the NGO centre helped them more than their schoolteachers. On the other hand, the privileged category students had access to quality teachers in their school.

**Box 1:** Verbatim extracts that substantiate supportive role of teacher

RP1 “[I always ask Bhaiya*^1^ at the centre. Sometimes to my schoolteacher, but Bhaiya always solves my doubts. My school does not have good teachers. I understand better at the centre]” RP5 [“I like Maths because my maths teacher at the centre really teaches well”] RP11 “I ask doubts from my teacher in the class itself” RP14 “Our science teacher tells us interesting stories and makes the subject so interesting” RP13 “I like history. I do not get bored in history class at all. Our teacher plays quizzes” RP2 “[My mother is not educated. I take help from my teacher at this centre for my doubts, and she always helps me].” [Other than studies, I like to dance and so I went to a dance teacher to learn dancing] RP7 “I used to go to school earlier, but now I only come to centre, because the teachers here are good” NGO Coordinator—“The volunteer teachers here are mostly engineers and are good at maths. Maybe that’s the reason many students like maths” **Memo** **Teachers who are supportive and helpful encourage students to engage with them at a personal level** **Involved Teachers try to generate interest for the subject** **Teachers who are approachable motivate students to study** **Children see teachers as quality experts who help them learn new things and solve their doubts. Children studying the NGO were getting access to quality teachers at the NGO centre and not in their school, unlike those from privileged families who acknowledged the quality teachers they learnt from in school**	

The Memo text recorded by the Authors during the research are highlighted in Bold

The underlined text in the box signify recrurring themes identified by paying attention to participant’s language

[] The translated verbatim extracts are enclosed in square brackets

^1^*Children in the NGO addressed the coordinator and the volunteer teachers there as “Bhaiya”, a term used to address elder brother in Hindi language

#### Parental support—involvement/support/help provided by parents to students for their studies

It is observed that children from both socio-economic categories see parents playing a positive supportive role in their studies. As can be seen from narratives captured in Box 2, educated parents help children with their doubts or learning process (RP13, RP10, RP14). Parents function as providers (RP2, RP6) and as motivators encouraging their child to achieve success (RP1). Parents play a supportive and encouraging role according to their capacity. As can be seen from RP1’s narrative extract in Box 2, despite being a single parent, who is not educated and is under financial pressure, the mother wants her son to be focused only on his studies. While this cannot be generalized and each child’s experience may vary depending on the family, we found a supportive role of parent being seen as a positive factor in the child’s school life. However, the experiences of children with parental support differed for children from underprivileged and privileged families. While for the underprivileged, all the participants believed their parents played a motivating role, but they were not educated and did not help them with studies. On the other hand, almost all the students from privileged families have their parents helping them with studies and learning, besides also motivating and providing for their needs.

**Box 2:** Verbatim extract that substantiate supportive role of parents

RP1 [“My mother is not educated. My father died when I was young. She wants me to focus only on my studies. I don’t work”] RP2 [“My mother buys me books for school”] RP6 [“My parents buy books and notebooks for me and whatever I need for studies”] RP13 “My father helps me with my studies. For my doubts I first ask my teacher in school or my father I have enrolled for online tuition classes in some subjects. The tutor sir helps me with my doubts.”” RP10 “normally I first ask my mom my doubts. Most of them are solved, only when she is not sure we check together on google.” RP12 “I go to my mother for clearing my doubts” **Memo** **Parents encourage children to achieve success** **Parents appreciation and involvement motivate the children** **Educated parents function as study buddies and help children with studies** **Parents function as providers to children**	

The Memo text recorded by the Authors during the research are highlighted in Bold

[] The translated verbatim extracts are enclosed in square brackets

#### Trusted study advisor—the person to whom the student goes for help with studies

Children from both Underprivileged and Privileged backgrounds had identified a trusted study advisor for themselves. This person was other than their schoolteachers and is their point of reference for help with school studies after school. This advisor could be an elder sibling, a parent, or a tuition teacher. As can be seen from Box 3 excerpts, the trusted study advisor may proactively help the student with exam preparations or regularly solve doubts when the child is not in school. Usually this is an individual so close to the child that he/she trusts fully and easy to approach. Interestingly, even the students who had access to apps and other technology solutions for study support, preferred to first ping their trusted study advisor. We found that while they used ICT device to learn many things of their interest by themselves, but for school studies, they preferred to reach their elder sibling, parent, or tuition teacher. Children from both categories had similar experience with respect to having a trusted study advisor.

**Box 3:** Verbatim extract that substantiate presence of a trusted study advisor

RP1 [“I always ask Bhaiya* at the centre. Sometimes to my teacher, but Bhaiya always solves my doubts”] RP13 “tuition sir helps me with my doubts” RP2 [“For studies I always take help from Bhaiya or my schoolteacher”] RP7 [“Bhaiya helps me with my studies and doubts”] RP9 [“Teacher in school taught me. I did not understand, I asked Bhaiya, and he helped me solve problems”] RP10 “For all subjects I take help from Mumma, for Spanish my elder brother helps me learn” RP11 “My father helps me before exams. I go to my dad when I get doubts while studying” **Memo** **An elder sibling, a parent, a tuition teacher can function as a trusted study advisor** **Children usually reach out to the trusted study advisor if they get stuck in studies** **Trusted study advisors may be proactively engaging with the child and help him/her with studies**	

The Memo text recorded by the Authors during the research are highlighted in Bold

[] The translated verbatim extracts are enclosed in square brackets

#### Self-directed learning-students identifying, planning, and executing their learning activities by own self

Children perceive that their own self-directed learning, interest, and motivation helps with their studies. Children from both backgrounds believe that they have a natural inclination for learning things of their interest by self-initiative. As seen in Box 4 excerpts, this is either triggered by a targeted achievement like high rank or scores in an exam, a career goal (RP2, RP1) or simply a learning curiosity (RP14, RP10, RP8). Experiences and perspective about self-directed learning that helped them to learn new things were similar for children from both categories.

**Box 4:** Verbatim extract that substantiate self-directed learning

RP1 [“I am preparing mental ability questions from a book myself; I like maths. I can learn myself from books, when I have doubts, I ask bhaiya for help. I enjoy both self-study and group study. I will become an engineer when I grow up.”] RP9 [“I study myself and I like maths and English.”] RP2 [“I want to be a doctor; I came first in the last exam. I study myself at home for 1.5 h daily] RP8 [“Last I learnt Divide in maths using YouTube video online by myself”] RP10 “I learnt making games online by myself. I did not take anyone’s help. But for school syllabus I only take help from my mother” RP12 “I have learnt many things by myself using internet online. I sometimes ask my brother or father if I get stuck installing something. I learnt video editing online by myself.” **Memo** **Children are naturally inclined to self-directed learning** **They learn things of their interest on their own** **They learn using ICT and apps** **They learn from books in solo as well as in groups** **Their self-direction, interest and motivation in learning helps with their studies**	

The Memo text recorded by the Authors during the research are highlighted in Bold

The underlined text in the box signify recrurring themes identified by paying attention to participant's language

[] The translated verbatim extracts are enclosed in square brackets

### Theme cluster: systemic/technology interventions that support school children with studies from their perspective

#### Affordability of device-low-cost mobiles/tablets/laptops/computers

Affordability of devices like mobile phone and computer surfaced as a key concern from the interactions with the children from underprivileged families as can be seen in the verbatim extracts captured in Box 5 from RP1, RP2, RP3, RP4 and RP5. This further corroborated with inputs provided by the NGO coordinator as shown in Box 5. However, this aspect was not observed anywhere in the narratives of children from privileged families. All interview participants from privileged families had their own personal computers/laptops/iPad (Refer narrative extracts from RP10, RP12, RP13, RP14). Experiences of children from both categories differed with respect to affordability of device. While children belonging to privileged could afford high-cost devices, those from underprivileged could not afford high-cost devices.

**Box 5:** Verbatim extract that substantiate affordability of device

RP1 [“I don’t have any device, nobody in my house has a mobile. My mother works as a house help, it is too expensive for us to buy”] RP2 [“We have only one mobile phone at home for all family members. I don’t have a computer….I have seen one in the school”] RP3 [“My father has a mobile phone. I use it sometimes to call Bhaiya”] RP4 [“I could not attend online classes. We don’t have a phone or computer”] RP5 [“No, I don’t have a mobile. My father has one, but I cannot attend classes in it”] NGO coordinator—“They live in nearby areas; their parents are daily wage workers. None of them have a mobile, their parents may have. Typically, in one family there may be one mobile.” RP10 “I have my own laptop” RP12 “I use my iPad” RP13 “I have a Personal Computer” RP14 “I have a laptop” **Memo** **Children who come from underprivileged families do not have access to devices like mobiles and laptops due to their cost. ICT devices must be affordable for all sections of the society or educational products must be innovated to work in all kinds of devices**	

The Memo text recorded by the Authors during the research are highlighted in Bold

The underlined text in the box signify recrurring themes identified by paying attention to participant's language

[] The translated verbatim extracts are enclosed in square brackets

#### Access to internet—availability of internet connection

Most of the participants from underprivileged families had no access to internet as they did not any device to access the same. On the other hand, while the children from privileged category participating in the study had internet access at home. Box 6 captures the verbatim extracts that substantiate this finding.

**Box 6:** Verbatim extract that substantiate access to internet

RP1 [“I have heard about apps and google, but never used, in my schoolteacher sometimes once in two months shows us the computer.”] RP2 [“I have never worked on internet”] RP3 [“I have used phone to access internet but not regularly. I once used it to learn tables and tenses when Bhaiya asked us to learn using phone.”] RP10 “I can access internet on my laptop” RP 13 “I have unlimited access to internet. I use my mother’s phone or my PC to connect on internet. I use it for three to four hours to play games or watching videos. I don’t use it for studies” RP14 “I have internet connection and I use internet for 1 h daily” **Memo** **Children from underprivileged hardly had access to internet while those from privileged had unlimited access**	

The Memo text recorded by the Authors during the research are highlighted in Bold

The underlined text in the box signify recrurring themes identified by paying attention to participant's language

[] The translated verbatim extracts are enclosed in square brackets

#### Mobile phone personal ownership

Since mobile phones are being considered as an alternative for low-cost study device, we checked with research participants from both category of families about personally owing a mobile device. If the device is not personally, children may not be able to use effectively if unavailable at the time required. As can be seen from Excerpts in Box 7 all the children in the sample did not own a mobile phone personally. It typically belonged to one of the family elders even if they had access to it. Experience with personal ownership of mobile phone was similar for children from both categories. This indicates that even in privileged families, parents rarely provide a personal mobile to their children while still in primary or secondary school.

**Box 7:** Verbatim extract that substantiate mobile phone device ownership

RP2 [“My mother has a smartphone; I have used it to play games.”] RP3 [“My father has a phone. With phone I attended an online class for 1 h in the morning during pandemic.”] RP4 [“I don’t have a phone. Sometimes I have seen my fathers”] RP7 [“My father has a mobile. I have seen cartoons sometimes in it, but I have not used it to study.”] RP11 “I don’t have a phone of my own. I use my mother’s” RP12 “No, I don’t have my own mobile. I have an iPad” RP14 “No, I don’t have a mobile of my own” **Memo** **Children in the sample do not personally own the mobile device while in school** **Usually, it belongs to a family elder and shared with the child. However, children from privileged families had alternate ICT devices personally owned by them like a laptop or an iPad that they could use at their disposal**	

The Memo text recorded by the Authors during the research are highlighted in Bold

[] The translated verbatim extracts are enclosed in square brackets

#### Financial sponsorship/aid

Fees for RP1 to RP9 was paid by the NGO and the children knew this when asked who paid their fees for school? On the other hand, for RP10 to RP14, parent paid the school fees. From the interactions children from privileged families seemed to be ignorant about fees and books, as it was made available to them by parents. Experience with financial sponsorship differed for both category children. Children in the NGO were getting a financial aid to pursue school. Despite enrolling in a municipal school, these children were being supported by volunteer teachers in the NGO and they regularly attended the classes at the NGO centre. On the other hand, the privileged category participants’ fee was paid by their parents, and they only attended their regular school (Box 8).

**Box 8:** Verbatim extract that substantiate financial sponsorship/aid

RP1 [“My fees are paid by the NGO Bhaiya. I like studies and the friends I get to make here. My school provides me mid-day meals”] RP2 [“Bhaiya gives me books and study materials in the centre. I have seen a computer in school. Sometimes the teachers show us “When asked how frequent, once in a week ?” once in two or three months”, “I want to be doctor”] RP8” [“I do not go to any other school. I used to but now I do not. I like this centre; they pay my fees”] RP13 “My parents pay my fees and buy all that I need for studies” **Memo** **Children from underprivileged recognized the financial support they were getting and were determined to work hard to improve their status. In fact, all of them had a career aspiration. However, the privileged category participants did not mention this aspect, upon inquiry recognized their parents as their funders or providers**	

The Memo text recorded by the Authors during the research are highlighted in Bold

[] The translated verbatim extracts are enclosed in square brackets

## Discussion

The purpose of this study was to explore the experiences of children and to develop a conceptual understanding of their perspective about studying for school and using technology for learning. We also examine how these experiences are distinct for students coming from underprivileged socio-economic backgrounds. Our proposed conceptual understanding reveals the key interventions according to the participants that support them with their studies for school. A total of eight themes under two clusters emerged from the present study. We integrate these themes with relevant literature in this section.

Table 2 below depicts the theme cluster categories that emerged from the analysis of data collected during the study.

**Table 2:** Theme cluster categories

Theme cluster	Sub-theme	Definition
Individual human intervention	Quality teacher	Person who teaches the subject in the institution
	Parental support	Involvement/support/encouragement of the parents towards the child's education
	Trusted study advisor	The person to whom the student goes for help with studies
	Self-directed learning	Students identifying, planning, and executing their learning activities by own self
Systemic/technological intervention	Affordability of device	Low-cost mobiles or tablets
	Access to internet	Internet with requisite bandwidth to support online education
	Mobile phone device personal ownership	A study device with focused educational content and internet access completely available at the student’s disposal
	Financial sponsorship/aid	Financial support to pursue education

**Fig. 1:**
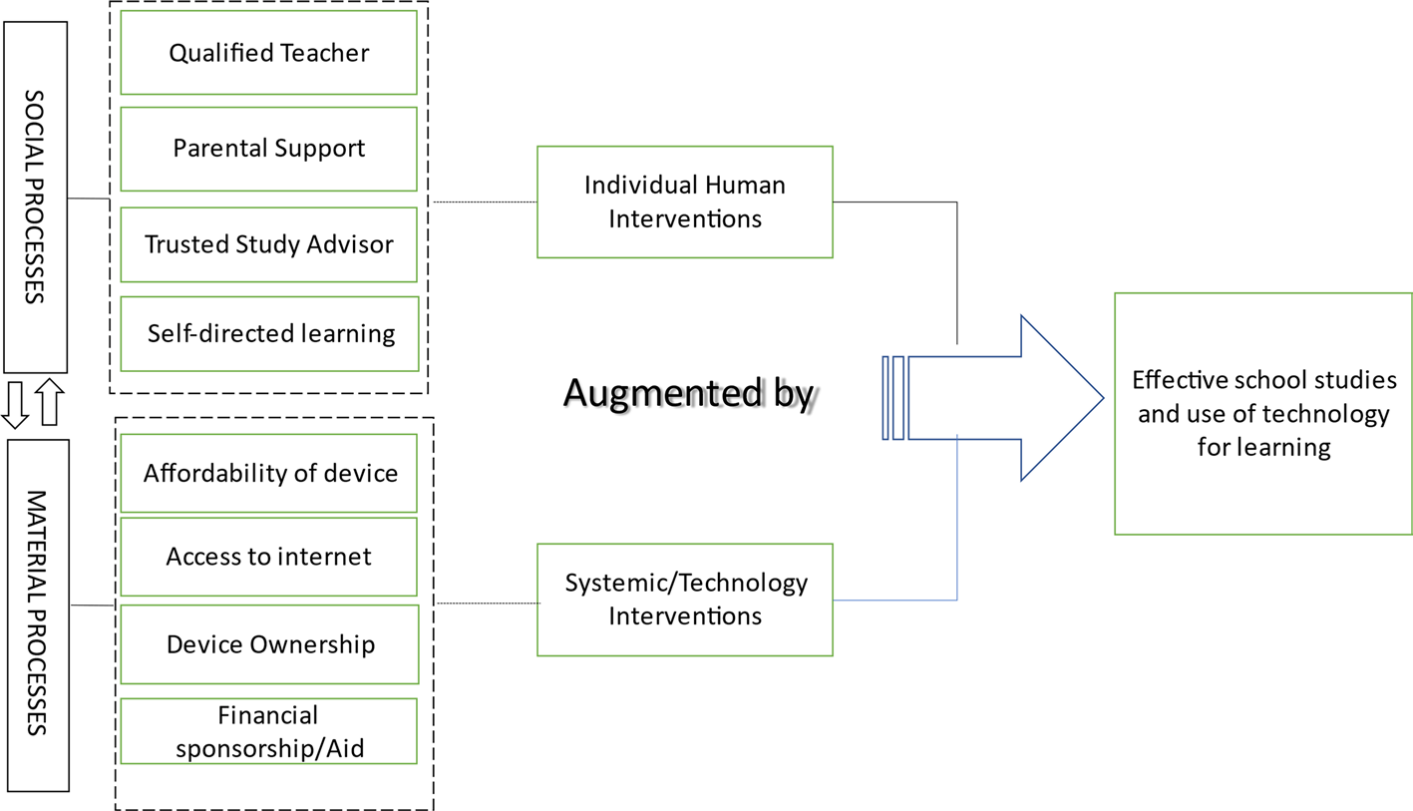
Framework to understand children’s perspective of school studies and use of technology

The proposed conceptual understanding reveals the challenges faced and strategies adopted by school children in school education and using technology for learning. Figure 1 depicts a conceptual framework to understand the children’s perspective about school studies and use of technology. Our finding is also in line with the concept of socio-materiality in adoption of technology. Social and the Material are entangled constitutively in multiple and dynamic ways in everyday life and must be addressed in technology adoption studies (Orlikowski, 2010).

The children belonging to the underprivileged families encountered challenges with affordability of educational device, access to internet, personal ownership of mobile device, financial support, and quality teacher at school. While the privileged background children did not face challenges related to affordability of device, access to internet and financial support, their experience with personal ownership of mobile device and quality teacher at school were like the experiences of the children from underprivileged background.

All the children adopted two key learning strategies. They were reaching out to their trusted study advisor when getting stuck with studies or they were pursuing self-directed learning, especially in areas of their own interest or to learn something new. However, children from privileged families were also reaching out to their parents for support with school studies. In fact, for most of them, one of their parents was their trusted study advisor. The reason why underprivileged students were not reaching out to parents for academic help was obvious, they recognized that their parents were not educated and could not help them with studies. Nevertheless, all the participating children from the underprivileged families claimed that their parents provided unconditional support to their education and encouraged them to focus only on studies.

The sub-themes in the present study, under individual human interventions and systemic/technological interventions, are identified from the participating children’s narratives and found to be influencing their school studies. In line with the constructivist grounded theory approach, we integrate these findings with previous studies related to school education and online learning in following paragraphs.

The need for quality teachers that emerged from this study echoes with several related studies in the past. Fisher (2017) has acknowledged that school-age students need access to trained educational professionals and resources. Many studies have discussed teacher education and development as a key reform and focus area for the government of India (Kumar & Azad, 2016; Kumar & Singh, n.d.; Mukherjee, 2014; Sharma, 2018; Walia, 2004).

Support and encouragement from parents came out in the discourses of all the children from both the cohorts of privileged and underprivileged. Despite their own low education status, the parents of all the participants from underprivileged background, provided unconditional support and encouragement to their wards for education. This may indicate that there is increased awareness among the poor, on the importance of education as the only means for the poor to alleviate poverty from their lives and their future generations. We found that children from both categories found parents playing a supporting role in their school education. Our finding is similar to findings from several other studies in the past related to the role of parents in school education (Desforges & Abouchaar, 2003; Fan & Chen, 2001; Goodall & Vorhaus, 2011; Harris & Goodall, 2008; Jeynes, 2007).

The participating children in this study from underprivileged families seldom used ICT for school studies because they did not have access to device as well as internet. Since their parents were not educated enough, they always reached out to their trusted study advisor for help with studies. Interestingly, despite awareness and access to ICT, the participants from privileged families were also found to be depended more on their parents, elder siblings, or tutors for help with school studies. While these children acknowledged that they used internet but all of them, interestingly, seldom used it for school studies. They, however, did claim to use ICT to learn areas of their personal interest. The present study therefore reveals that children prefer to reach out to a trusted study advisor irrespective of their access to ICT tools and apps. Advanced apps are underway to make learning an autonomous activity, we infer from this study that the role of trusted study advisors must be more closely examined and incorporated as these progressions happen. Borup and Drysdale (2014) also highlighted in their study that school students lack meta-cognitive ability and self-regulation ability to succeed in a highly autonomous learning environment and require auxiliary support from a facilitator whose role may involve fostering the relationship, monitoring, and instructing. While they are talking about facilitators for effective online learning, we recommend the concept of trusted study advisors must be explored at a broader level. Especially for students coming from underprivileged family backgrounds, who may not have educated family members, relatives, or friends to help them with studies, it becomes imperative to consider developing a community of voluntary study mentors in the society.

Self-directed learning emerged a key learning strategy of participants from both cohorts. While it was evident in the form of organizing, scheduling, and planning for studies among underprivileged participants, for others it was learning using internet and learning apps, particularly in areas of their interest. A recent study has similar findings that self-testing, scheduling and concept maps created by students positively correlate with student’s academic performance (Xu et al., 2021) and students who adopt self-direction achieve better academic success. Self-directed learning is a twenty-first century skill and efforts are already underway to inculcate the same in children while they are in school to prepare them for their future (Bartholomew et al., 2017; Mentz et al., 2021; Voskamp et al., 2020). The finding from the present research affirms the children’s perspective towards pursuing self-directed learning in areas that interests them. This corroborates with the efforts underway towards understanding learner interests and hyper-personalization of online learning offerings by EdTech firms.

Affordability of the study device was found as a key concern for underprivileged students in the present study. Widespread success of the ICT enabled school education requires significant focus towards availability of low-cost devices. The presence of a digital divide in India owing to socio-economic status differences is highlighted in several studies (Hillier, 2018; Kumar & Kumara, 2018; Rao, 2005; Tewathia et al., 2020; Venkatesh & Sykes, 2013). Fisher (2017) has also found that school aged children need access to resources irrespective of their socio-economic status. Therefore, in order that online learning is leveraged fully to provide quality school education, especially for students in remote locations, efforts are required to reduce the cost of devices. Focus on rugged, low-cost equipment designed for sustainable use in local environment is very important to serve rural learners (Ramani, 2015).

Just like devices children from underprivileged families also lack access to internet. Bakia et al. (2012) in their study have also said that broadening access to dramatically reduce the cost of providing access to quality educational resources and experiences particularly for students in remote locations is key to leverage online learning.

Interestingly participants from both cohorts in the sample, did not own mobile phone device personally for unlimited time. They used the mobile phones of their parents for limited time on need basis. Unlike children from privileged families who had access to other ICT study devices like a computer, laptop, or iPad, those from underprivileged families had no access to any ICT device. Therefore, while not owning a personal mobile may not a challenge at this time for privileged students, it is one for the underprivileged. With increased thrust towards using mobile based solutions for increased affordability and reach, it becomes imperative from this finding to examine the ownership aspect in greater detail. Not all schools in India still allow students to carry mobile phones. Not all parents in India are still open to give mobile phones to their children. In this context, it may be useful for EdTech firms to develop solutions and devices that can be particularly used only for school studies and are affordable for students from underprivileged families.

All the participants from the underprivileged families were getting financial aid for their school education. While there are many government schemes and programs that support education for underprivileged children, we suggest that the financial sponsorships and aid must be revisited to enhance their quality of educational experience. Fisher (2017) has also pointed out that there is a need for policies and regulations to provide quality educational opportunities regardless of socio-economic status of the children. Several other studies have also insisted the importance of examining financial support/aid for school education to provide equal educational experiences irrespective of socio-economic (Malvankar, 2018; Purwant, 2020; Rao, 2004; Tyagi, 2014; Yi et al., 2015).

While we found significant work happening around most of the themes identified in the present study, the areas of trusted study advisors, personal ownership or mobile phone, and self-directed learning by students only in areas of their personal interest are relatively new. We believe, these new themes along with the conceptual framework to understand school learning in the perspective of children, are contributions of the present research for enhancing literature in school education and online learning. Smart Learning Environment implies the practical coalescence of different elements of educational curriculum, enriched methodologies and strategies, enriched assessment, educational roles, smart technology and a symbiotic presence of both physical and virtual environment (García-Tudela et al., 2021). The new themes identified from the present study may further contribute to enhancing the learning outcomes for school students from these smart environments being envisaged for school education.

## Research implications of the study

Past studies have recognized technology as promising solution to implementing universal school education across the world. Studies have also addressed the challenges to implement this in India due to the digital and socio-economic divide. At the same time, previous studies have also captured the government initiatives to address the digital divide and enabling technology adoption for school education. Case based and experimental research has been undertaken and found that capacity of children to adopt technology for learning exists irrespective of their family backgrounds. In this study, we add to the existing literature by capturing children’s perspective on school education and learning into a theoretical framework. We verify findings from previous research by corroborating it with the perspective of the primary stakeholders and consumers of these initiatives namely the children and identify new themes (Trusted study advisors, Self-directed learning, and Personal ownership of device) for future exploration in this area. The present study also brings out the distinct experiences, challenges and learning strategies of children from privileged and underprivileged families. The findings and the conceptual model can be further validated with different student samples.

## Practical implications of the study

The need to promote equitable education and learning for social and economic growth is unquestionable. The advent of mobile phones presents a wonderful opportunity and offers a timely challenge to re-define and transform educational paradigms. However, the findings from this study captures the students’ perspective (who are at the receiving end of these initiatives) of how they study and use technology for the same. The framework we have proposed may be used by EdTech providers and policy makers while building strategies for universal school education in the country. Many of our findings are in line with findings in previous studies and actions are already underway to leverage them like initiatives to bridge the digital divide, enhancing financial aid and increasing recruitment of quality teachers as discussed in the literature review. We identify the unique and critical role of a “trusted study advisor” in every school student’s life. Students from underprivileged students, may not have educated parents or family members to support them with studies and the number of quality teachers in schools they study are low. Retired professionals may volunteer to mentor by becoming their trusted study advisors. States may encourage “Each one Mentor one” programs to enhance the quality of support for children belonging to underprivileged families. EdTech firms may review their products and work on offerings that provides affordable study devices to children which they have at their disposal for learning like the Byju’s tablet (Tripathy & Devarapalli, 2021). Educational pedagogies may be revisited to leverage the innate self-directed learning tendencies of children to enhance their educational outcomes. Government initiatives around ensuring connectedness are already underway, however equal focus needs to be given to training and producing more quality teachers and improve the quality of school education in aided schools. Implementation of smart learning projects in public schools changes the role of teachers from primary source of information to a facilitator, guide and coach (Khlaif & Farid, 2018). Teacher’s training institutions may work to strengthen the desired behaviours of education deliverers. Education sponsoring agencies may be more mindful to hire the right teachers and increase support to provide required infrastructure. Remote learning content creators may provide content to fill the gap of quality teachers.

## Conclusion

The vision of universal education and 100% literacy that we have laid out in the national education policy-2020, calls for some tangible high impact actions. The present study adds to existing literature by capturing the student’s perspective and the distinct experiences of the children from underprivileged backgrounds. We found that the experiences of privileged and underprivileged children differed with respect to access to internet, affordability of ICT device, quality teachers, parental support, and financial sponsorship. However, the experiences and perspectives of the children were found to be similar with respect to personal ownership of ICT device for unlimited time, pursuing self-directed learning and having a trusted study advisor. The findings may be useful to policy makers and EdTech firms to build strategies and solutions for effective implementation of universal school education in the country.

## Limitations and future scope of the study

The study was conducted in northern India and can be validated further with different student samples from other parts of the country. Constructivist grounded theorists see meaning as mutually constructed between the researcher and the researched. Therefore, we do not claim any neutrality in our process of making meaning here, however we have tried our best to use rigorous data analysis strategies and to report on these. The data for the research was collected by respecting the agency of the children as active participants in interpreting their own world and particular care was taken to keep the questions open-ended so that the children can raise and talk about the issues that are important to them. The results are drawn from interactions with a small sample of children. It is recommended that these findings be further validated with larger and varied samples of school students. The framework and the new themes of “Trusted study advisor”, “Self-directed learning”, and “Personal ownership of mobile device” for children, can be explored in future studies.

## Data Availability

The datasets used and/or analysed during the current study are available from the corresponding author on reasonable request.

## Abbreviations

EdTech: Educational Technology
Govt.: Government
ICT: Internet and Communication Technology
Mgmnt.: Management
NCR: National Capital Region
NGO: Non-Governmental Organization
RP: Research participant
